# Supplementary material for: Pyruvate Dehydrogenase Kinase 1 inhibition mediated oxidative phosphorylation enhancement in cartilage promotes osteoarthritis progression
Source: BMC Musculoskelet Disord. 2023 Jul 20;24:597. doi: 10.1186/s12891-023-06585-6 (PMC10357736; doi:10.1186/s12891-023-06585-6)
Supplement: Supplementary file 1 — Additional file 1. [file 12891_2023_6585_MOESM1_ESM.zip › KEGG.Gsea.1653622432464/KEGG_OOCYTE_MEIOSIS.html]

Details for gene set KEGG\_OOCYTE\_MEIOSIS[GSEA]

|  || Dataset | 55457\_collapsed\_to\_symbols.cls.cls #OA\_versus\_normal.cls.cls #OA\_versus\_normal\_repos |
| Phenotype | cls.cls#OA\_versus\_normal\_repos |
| Upregulated in class | OA |
| GeneSet | KEGG\_OOCYTE\_MEIOSIS |
| Enrichment Score (ES) | 0.16446525 |
| Normalized Enrichment Score (NES) | 0.76284057 |
| Nominal p-value | 0.9478022 |
| FDR q-value | 0.9923012 |
| FWER p-Value | 1.0 |
Table: GSEA Results Summary

  

Fig 1: Enrichment plot: KEGG\_OOCYTE\_MEIOSIS      
 Profile of the Running ES Score & Positions of GeneSet Members on the Rank Ordered List

  

| SYMBOL | TITLE | RANK IN GENE LIST | RANK METRIC SCORE | RUNNING ES | CORE ENRICHMENT || 1 | YWHAH | tyrosine 3-monooxygenase/tryptophan 5-monooxygenase activation protein eta [Source:HGNC Symbol;Acc:HGNC:12853] | 118 | 0.889 | 0.0214 | Yes |
| 2 | RBX1 | ring-box 1 [Source:HGNC Symbol;Acc:HGNC:9928] | 348 | 0.724 | 0.0284 | Yes |
| 3 | CDC20 | cell division cycle 20 [Source:HGNC Symbol;Acc:HGNC:1723] | 552 | 0.641 | 0.0346 | Yes |
| 4 | PPP1CA | protein phosphatase 1 catalytic subunit alpha [Source:HGNC Symbol;Acc:HGNC:9281] | 603 | 0.622 | 0.0521 | Yes |
| 5 | PPP1CC | protein phosphatase 1 catalytic subunit gamma [Source:HGNC Symbol;Acc:HGNC:9283] | 744 | 0.583 | 0.0612 | Yes |
| 6 | MAPK3 | mitogen-activated protein kinase 3 [Source:HGNC Symbol;Acc:HGNC:6877] | 757 | 0.579 | 0.0802 | Yes |
| 7 | PRKACB | protein kinase cAMP-activated catalytic subunit beta [Source:HGNC Symbol;Acc:HGNC:9381] | 771 | 0.576 | 0.0990 | Yes |
| 8 | CAMK2A | calcium/calmodulin dependent protein kinase II alpha [Source:HGNC Symbol;Acc:HGNC:1460] | 916 | 0.538 | 0.1062 | Yes |
| 9 | CALM3 | calmodulin 3 [Source:HGNC Symbol;Acc:HGNC:1449] | 1039 | 0.510 | 0.1142 | Yes |
| 10 | ANAPC13 | anaphase promoting complex subunit 13 [Source:HGNC Symbol;Acc:HGNC:24540] | 1116 | 0.493 | 0.1253 | Yes |
| 11 | CALML5 | calmodulin like 5 [Source:HGNC Symbol;Acc:HGNC:18180] | 1261 | 0.465 | 0.1300 | Yes |
| 12 | PTTG1 | "PTTG1 regulator of sister chromatid separation, securin [Source:HGNC Symbol;Acc:HGNC:9690]" | 1329 | 0.453 | 0.1404 | Yes |
| 13 | ADCY7 | adenylate cyclase 7 [Source:HGNC Symbol;Acc:HGNC:238] | 1408 | 0.442 | 0.1495 | Yes |
| 14 | MAD2L1 | mitotic arrest deficient 2 like 1 [Source:HGNC Symbol;Acc:HGNC:6763] | 1516 | 0.427 | 0.1558 | Yes |
| 15 | ADCY1 | adenylate cyclase 1 [Source:HGNC Symbol;Acc:HGNC:232] | 1589 | 0.415 | 0.1645 | Yes |
| 16 | PPP2R5D | protein phosphatase 2 regulatory subunit B'delta [Source:HGNC Symbol;Acc:HGNC:9312] | 2137 | 0.346 | 0.1336 | No |
| 17 | IGF1 | insulin like growth factor 1 [Source:HGNC Symbol;Acc:HGNC:5464] | 2286 | 0.328 | 0.1334 | No |
| 18 | CCNB1 | cyclin B1 [Source:HGNC Symbol;Acc:HGNC:1579] | 2359 | 0.319 | 0.1387 | No |
| 19 | BUB1 | BUB1 mitotic checkpoint serine/threonine kinase [Source:HGNC Symbol;Acc:HGNC:1148] | 2388 | 0.315 | 0.1474 | No |
| 20 | CCNE2 | cyclin E2 [Source:HGNC Symbol;Acc:HGNC:1590] | 2469 | 0.306 | 0.1517 | No |
| 21 | PPP2R5A | protein phosphatase 2 regulatory subunit B'alpha [Source:HGNC Symbol;Acc:HGNC:9309] | 2592 | 0.291 | 0.1522 | No |
| 22 | STAG3 | stromal antigen 3 [Source:HGNC Symbol;Acc:HGNC:11356] | 2761 | 0.274 | 0.1485 | No |
| 23 | CAMK2G | calcium/calmodulin dependent protein kinase II gamma [Source:HGNC Symbol;Acc:HGNC:1463] | 2900 | 0.261 | 0.1467 | No |
| 24 | ESPL1 | "extra spindle pole bodies like 1, separase [Source:HGNC Symbol;Acc:HGNC:16856]" | 3122 | 0.238 | 0.1376 | No |
| 25 | PPP2R5C | protein phosphatase 2 regulatory subunit B'gamma [Source:HGNC Symbol;Acc:HGNC:9311] | 3205 | 0.230 | 0.1391 | No |
| 26 | RPS6KA1 | ribosomal protein S6 kinase A1 [Source:HGNC Symbol;Acc:HGNC:10430] | 3389 | 0.213 | 0.1322 | No |
| 27 | PLK1 | polo like kinase 1 [Source:HGNC Symbol;Acc:HGNC:9077] | 3556 | 0.198 | 0.1260 | No |
| 28 | CDC23 | cell division cycle 23 [Source:HGNC Symbol;Acc:HGNC:1724] | 3729 | 0.183 | 0.1189 | No |
| 29 | CDK1 | cyclin dependent kinase 1 [Source:HGNC Symbol;Acc:HGNC:1722] | 3744 | 0.182 | 0.1240 | No |
| 30 | PPP3CA | protein phosphatase 3 catalytic subunit alpha [Source:HGNC Symbol;Acc:HGNC:9314] | 3803 | 0.176 | 0.1256 | No |
| 31 | INS | insulin [Source:HGNC Symbol;Acc:HGNC:6081] | 3885 | 0.166 | 0.1249 | No |
| 32 | PPP2CA | protein phosphatase 2 catalytic subunit alpha [Source:HGNC Symbol;Acc:HGNC:9299] | 4146 | 0.143 | 0.1095 | No |
| 33 | ITPR2 | "inositol 1,4,5-trisphosphate receptor type 2 [Source:HGNC Symbol;Acc:HGNC:6181]" | 4222 | 0.135 | 0.1083 | No |
| 34 | CDC16 | cell division cycle 16 [Source:HGNC Symbol;Acc:HGNC:1720] | 4566 | 0.108 | 0.0853 | No |
| 35 | PTTG2 | pituitary tumor-transforming 2 [Source:HGNC Symbol;Acc:HGNC:9691] | 4621 | 0.103 | 0.0846 | No |
| 36 | AURKA | aurora kinase A [Source:HGNC Symbol;Acc:HGNC:11393] | 4916 | 0.079 | 0.0643 | No |
| 37 | SMC1A | structural maintenance of chromosomes 1A [Source:HGNC Symbol;Acc:HGNC:11111] | 4942 | 0.077 | 0.0650 | No |
| 38 | ADCY9 | adenylate cyclase 9 [Source:HGNC Symbol;Acc:HGNC:240] | 5191 | 0.057 | 0.0476 | No |
| 39 | PPP3CB | protein phosphatase 3 catalytic subunit beta [Source:HGNC Symbol;Acc:HGNC:9315] | 5311 | 0.046 | 0.0399 | No |
| 40 | ANAPC10 | anaphase promoting complex subunit 10 [Source:HGNC Symbol;Acc:HGNC:24077] | 5424 | 0.039 | 0.0325 | No |
| 41 | CCNE1 | cyclin E1 [Source:HGNC Symbol;Acc:HGNC:1589] | 5663 | 0.018 | 0.0145 | No |
| 42 | YWHAZ | tyrosine 3-monooxygenase/tryptophan 5-monooxygenase activation protein zeta [Source:HGNC Symbol;Acc:HGNC:12855] | 5770 | 0.010 | 0.0066 | No |
| 43 | CALML3 | calmodulin like 3 [Source:HGNC Symbol;Acc:HGNC:1452] | 6348 | -0.032 | -0.0374 | No |
| 44 | CPEB1 | cytoplasmic polyadenylation element binding protein 1 [Source:HGNC Symbol;Acc:HGNC:21744] | 6435 | -0.038 | -0.0428 | No |
| 45 | ADCY8 | adenylate cyclase 8 [Source:HGNC Symbol;Acc:HGNC:239] | 6646 | -0.053 | -0.0574 | No |
| 46 | ANAPC5 | anaphase promoting complex subunit 5 [Source:HGNC Symbol;Acc:HGNC:15713] | 6751 | -0.059 | -0.0634 | No |
| 47 | ITPR1 | "inositol 1,4,5-trisphosphate receptor type 1 [Source:HGNC Symbol;Acc:HGNC:6180]" | 6752 | -0.059 | -0.0614 | No |
| 48 | ANAPC2 | anaphase promoting complex subunit 2 [Source:HGNC Symbol;Acc:HGNC:19989] | 6787 | -0.062 | -0.0619 | No |
| 49 | MAPK1 | mitogen-activated protein kinase 1 [Source:HGNC Symbol;Acc:HGNC:6871] | 6853 | -0.068 | -0.0647 | No |
| 50 | SMC3 | structural maintenance of chromosomes 3 [Source:HGNC Symbol;Acc:HGNC:2468] | 6950 | -0.076 | -0.0696 | No |
| 51 | PPP3CC | protein phosphatase 3 catalytic subunit gamma [Source:HGNC Symbol;Acc:HGNC:9316] | 7031 | -0.082 | -0.0730 | No |
| 52 | RPS6KA3 | ribosomal protein S6 kinase A3 [Source:HGNC Symbol;Acc:HGNC:10432] | 7092 | -0.087 | -0.0747 | No |
| 53 | PPP2CB | protein phosphatase 2 catalytic subunit beta [Source:HGNC Symbol;Acc:HGNC:9300] | 7265 | -0.100 | -0.0847 | No |
| 54 | FBXO5 | F-box protein 5 [Source:HGNC Symbol;Acc:HGNC:13584] | 8007 | -0.156 | -0.1372 | No |
| 55 | RPS6KA6 | ribosomal protein S6 kinase A6 [Source:HGNC Symbol;Acc:HGNC:10435] | 8071 | -0.161 | -0.1366 | No |
| 56 | MAPK12 | mitogen-activated protein kinase 12 [Source:HGNC Symbol;Acc:HGNC:6874] | 8161 | -0.168 | -0.1377 | No |
| 57 | AR | androgen receptor [Source:HGNC Symbol;Acc:HGNC:644] | 8217 | -0.173 | -0.1361 | No |
| 58 | RPS6KA2 | ribosomal protein S6 kinase A2 [Source:HGNC Symbol;Acc:HGNC:10431] | 8397 | -0.185 | -0.1437 | No |
| 59 | SKP1 | S-phase kinase associated protein 1 [Source:HGNC Symbol;Acc:HGNC:10899] | 8454 | -0.190 | -0.1415 | No |
| 60 | ADCY3 | adenylate cyclase 3 [Source:HGNC Symbol;Acc:HGNC:234] | 8572 | -0.199 | -0.1438 | No |
| 61 | CHP2 | calcineurin like EF-hand protein 2 [Source:HGNC Symbol;Acc:HGNC:24927] | 8573 | -0.199 | -0.1369 | No |
| 62 | YWHAQ | tyrosine 3-monooxygenase/tryptophan 5-monooxygenase activation protein theta [Source:HGNC Symbol;Acc:HGNC:12854] | 8675 | -0.207 | -0.1377 | No |
| 63 | CAMK2B | calcium/calmodulin dependent protein kinase II beta [Source:HGNC Symbol;Acc:HGNC:1461] | 8782 | -0.215 | -0.1386 | No |
| 64 | CDC27 | cell division cycle 27 [Source:HGNC Symbol;Acc:HGNC:1728] | 8944 | -0.227 | -0.1434 | No |
| 65 | PGR | progesterone receptor [Source:HGNC Symbol;Acc:HGNC:8910] | 9048 | -0.235 | -0.1433 | No |
| 66 | MOS | "MOS proto-oncogene, serine/threonine kinase [Source:HGNC Symbol;Acc:HGNC:7199]" | 9138 | -0.243 | -0.1419 | No |
| 67 | SLK | STE20 like kinase [Source:HGNC Symbol;Acc:HGNC:11088] | 9362 | -0.261 | -0.1503 | No |
| 68 | CDC25C | cell division cycle 25C [Source:HGNC Symbol;Acc:HGNC:1727] | 9501 | -0.274 | -0.1517 | No |
| 69 | PPP2R5B | protein phosphatase 2 regulatory subunit B'beta [Source:HGNC Symbol;Acc:HGNC:9310] | 9554 | -0.279 | -0.1462 | No |
| 70 | ANAPC1 | anaphase promoting complex subunit 1 [Source:HGNC Symbol;Acc:HGNC:19988] | 9630 | -0.286 | -0.1422 | No |
| 71 | ADCY2 | adenylate cyclase 2 [Source:HGNC Symbol;Acc:HGNC:233] | 9715 | -0.293 | -0.1387 | No |
| 72 | CUL1 | cullin 1 [Source:HGNC Symbol;Acc:HGNC:2551] | 9762 | -0.297 | -0.1320 | No |
| 73 | CALM1 | calmodulin 1 [Source:HGNC Symbol;Acc:HGNC:1442] | 10136 | -0.334 | -0.1497 | No |
| 74 | MAP2K1 | mitogen-activated protein kinase kinase 1 [Source:HGNC Symbol;Acc:HGNC:6840] | 10197 | -0.340 | -0.1426 | No |
| 75 | ITPR3 | "inositol 1,4,5-trisphosphate receptor type 3 [Source:HGNC Symbol;Acc:HGNC:6182]" | 10390 | -0.360 | -0.1452 | No |
| 76 | PPP2R5E | protein phosphatase 2 regulatory subunit B'epsilon [Source:HGNC Symbol;Acc:HGNC:9313] | 10392 | -0.360 | -0.1329 | No |
| 77 | PPP2R1A | protein phosphatase 2 scaffold subunit Aalpha [Source:HGNC Symbol;Acc:HGNC:9302] | 10395 | -0.360 | -0.1207 | No |
| 78 | PRKACG | protein kinase cAMP-activated catalytic subunit gamma [Source:HGNC Symbol;Acc:HGNC:9382] | 10466 | -0.370 | -0.1135 | No |
| 79 | PPP2R1B | protein phosphatase 2 scaffold subunit Abeta [Source:HGNC Symbol;Acc:HGNC:9303] | 10578 | -0.382 | -0.1090 | No |
| 80 | CALM2 | calmodulin 2 [Source:HGNC Symbol;Acc:HGNC:1445] | 10579 | -0.382 | -0.0959 | No |
| 81 | PRKACA | protein kinase cAMP-activated catalytic subunit alpha [Source:HGNC Symbol;Acc:HGNC:9380] | 10611 | -0.384 | -0.0851 | No |
| 82 | CCNB2 | cyclin B2 [Source:HGNC Symbol;Acc:HGNC:1580] | 10639 | -0.387 | -0.0739 | No |
| 83 | BTRC | beta-transducin repeat containing E3 ubiquitin protein ligase [Source:HGNC Symbol;Acc:HGNC:1144] | 10836 | -0.409 | -0.0751 | No |
| 84 | CHP1 | calcineurin like EF-hand protein 1 [Source:HGNC Symbol;Acc:HGNC:17433] | 10940 | -0.423 | -0.0686 | No |
| 85 | REC8 | REC8 meiotic recombination protein [Source:HGNC Symbol;Acc:HGNC:16879] | 11016 | -0.432 | -0.0596 | No |
| 86 | CDK2 | cyclin dependent kinase 2 [Source:HGNC Symbol;Acc:HGNC:1771] | 11069 | -0.441 | -0.0485 | No |
| 87 | PKMYT1 | "protein kinase, membrane associated tyrosine/threonine 1 [Source:HGNC Symbol;Acc:HGNC:29650]" | 11403 | -0.487 | -0.0577 | No |
| 88 | YWHAB | tyrosine 3-monooxygenase/tryptophan 5-monooxygenase activation protein beta [Source:HGNC Symbol;Acc:HGNC:12849] | 11488 | -0.500 | -0.0471 | No |
| 89 | PRKX | protein kinase X-linked [Source:HGNC Symbol;Acc:HGNC:9441] | 11634 | -0.522 | -0.0404 | No |
| 90 | PPP1CB | protein phosphatase 1 catalytic subunit beta [Source:HGNC Symbol;Acc:HGNC:9282] | 11737 | -0.539 | -0.0298 | No |
| 91 | FBXW11 | F-box and WD repeat domain containing 11 [Source:HGNC Symbol;Acc:HGNC:13607] | 11982 | -0.590 | -0.0286 | No |
| 92 | ADCY6 | adenylate cyclase 6 [Source:HGNC Symbol;Acc:HGNC:237] | 12005 | -0.594 | -0.0099 | No |
| 93 | YWHAE | tyrosine 3-monooxygenase/tryptophan 5-monooxygenase activation protein epsilon [Source:HGNC Symbol;Acc:HGNC:12851] | 12137 | -0.628 | 0.0015 | No |
| 94 | PPP3R1 | "protein phosphatase 3 regulatory subunit B, alpha [Source:HGNC Symbol;Acc:HGNC:9317]" | 12653 | -0.829 | -0.0102 | No |
| 95 | IGF1R | insulin like growth factor 1 receptor [Source:HGNC Symbol;Acc:HGNC:5465] | 12689 | -0.855 | 0.0165 | No |
Table: GSEA details [plain text format]

  

Fig 2: KEGG\_OOCYTE\_MEIOSIS      
 Blue-Pink O' Gram in the Space of the Analyzed GeneSet

  

Fig 3: KEGG\_OOCYTE\_MEIOSIS: Random ES distribution      
 Gene set null distribution of ES for **KEGG\_OOCYTE\_MEIOSIS**
